# Supplementary material for: Profile of risk factors for Non-Communicable Diseases (NCDs) in a highly urbanized district of India: Findings from Puducherry district-wide STEPS Survey, 2019–20
Source: PLoS One. 2021 Jan 12;16(1):e0245254. doi: 10.1371/journal.pone.0245254 (PMC7802941; doi:10.1371/journal.pone.0245254)
Supplement: Attachment — Submitted filename: PONE-D-20-21171.pdf [file pone.0245254.s001.pdf]

PLOS ONE

Profile of risk factors for Non-Communicable Diseases (NCDs) in a highly urbanized district of India: Findings from district-wide STEPS Survey

--Manuscript Draft--

Manuscript Number:	PONE-D-20-21171
Article Type:	Research Article
Full Title:	Profile of risk factors for Non-Communicable Diseases (NCDs) in a highly urbanized district of India: Findings from district-wide STEPS Survey
Short Title:	NCD Risk Factors profile in Puducherry district, India
Corresponding Author:	Sitanshu Sekhar Kar, MD<br>JIPMER<br>Puducherry, Puducherry INDIA
Keywords:	Non Communicable Diseases, Risk factors, WHO STEPS, Hypertension, Diabetes, Obesity, INDIA
Abstract:	Introduction: Rapid urbanization and industrialization drives the rising burden of Non-Communicable Diseases (NCDs) worldwide that are characterized by uptake of unhealthy lifestyle such as tobacco and alcohol use, physical inactivity and unhealthy diet. In India, the prevalence of various NCDs and its risk factors shows wide variations across geographic regions necessitating region-specific evidence for population-based prevention and control of NCDs.<br>Objective: To estimate the prevalence of behavioral and biological risk factors of NCDs among adult population (18-69 years) in the district of Puducherry in South India.<br>Methodology: World Health Organization (WHO) prescribed STEPwise approach to NCD surveillance (STEPS) was conducted by following all three steps (behavioral, physical and biochemical measurements). A total of 2560 individuals were selected from urban and rural areas (50 clusters in each) through multi-stage cluster random sampling method. STEPS instrument was used to assess behavioral and physical measurements. Fasting blood sample was collected to estimate biochemical risk factors (Diabetes, Hypercholesterolemia) of NCDs.<br>Results: Among men, alcohol use 40.4% (95% CI: 37.4-43.4) was almost twice higher compared to tobacco use 24.4% (95% CI: 21.7-26.9). Nearly half of the population was physically inactive 45.8% (95% CI: 43.8-47.8) and obese 46.1% (95% CI: 44-48.1). Hypertension and diabetes mellitus was present among one-third 33.6% (95% CI: 31.6-35.5) and one-fourth 26.7% (95% CI: 24.1-29.1) of the population which were significantly higher among men. Physical inactivity and overweight/obesity increased with increasing education levels. Tobacco and alcohol use was more common among men, whereas physical inactivity with obesity and hypercholesterolemia was higher among women.<br>Conclusion: The study highlights high prevalence of various NCDs and its risk factors among the adult population of Puducherry district. Population based health promotion interventions need to be initiated with major emphasis on the urban population of the district.
Order of Authors:	Parthibane Sivanantham
	Subitha Lakshminarayanan
	Jayaprakash Sahoo
	Zachariah Bobby
	Sitanshu Sekhar Kar, MD
Opposed Reviewers:	
Additional Information:	
**Question**	**Response**
**Financial Disclosure**	Dr Sitanshu Sekhar Kar received the grant for the study.

Powered by Editorial Manager® and ProduXion Manager® from Aries Systems Corporation

Enter a financial disclosure statement that describes the sources of funding for the work included in this submission. Review the submission guidelines for detailed requirements. View published research articles from *PLOS ONE* for specific examples.<br><br>This statement is required for submission and **will appear in the published article** if the submission is accepted. Please make sure it is accurate.<br><br>**Unfunded studies**<br>Enter: *The author(s) received no specific funding for this work.*<br><br>**Funded studies**<br>Enter a statement with the following details:<br>• Initials of the authors who received each award<br>• Grant numbers awarded to each author<br>• The full name of each funder<br>• URL of each funder website<br>• Did the sponsors or funders play any role in the study design, data collection and analysis, decision to publish, or preparation of the manuscript?<br>• **NO** - Include this sentence at the end of your statement: *The funders had no role in study design, data collection and analysis, decision to publish, or preparation of the manuscript.*<br>• **YES** - Specify the role(s) played.<br><br>* typeset	This work was funded by the Jawaharlal Institute of Postgraduate Medical Education and Research (JIPMER)<br><br>Grant No. JIP/Dean (R)/Intramural/Phs 1/2019-20<br><br>The url of Funder website: jipmer.edu.in/<br><br>The funder did not have any role in study design, data collection and analysis, decision to publish or preparation of manuscript
**Competing Interests**<br><br>Use the instructions below to enter a competing interest statement for this submission. On behalf of all authors, disclose any competing interests that could be perceived to bias this work—acknowledging all financial support and any other relevant financial or non-financial competing interests.<br><br>This statement **will appear in the published article** if the submission is accepted. Please make sure it is accurate. View published research articles from *PLOS ONE* for specific examples.	The authors have declared that no competing interests exist.

Powered by Editorial Manager® and ProduXion Manager® from Aries Systems Corporation

**NO authors have competing interests**<br><br>Enter: *The authors have declared that no competing interests exist.*<br><br>**Authors with competing interests**<br><br>Enter competing interest details beginning with this statement:<br><br>*I have read the journal's policy and the authors of this manuscript have the following competing interests: [insert competing interests here]*<br><br>* typeset	
**Ethics Statement**<br><br>Enter an ethics statement for this submission. This statement is required if the study involved:<br><br>• Human participants<br>• Human specimens or tissue<br>• Vertebrate animals or cephalopods<br>• Vertebrate embryos or tissues<br>• Field research<br><br>Write "N/A" if the submission does not require an ethics statement.<br><br>General guidance is provided below. Consult the submission guidelines for detailed instructions. **Make sure that all information entered here is included in the Methods section of the manuscript.**	The study was approved by 'JIPMER Institutional Ethics Committee (Human Studies)'<br><br>Approval Number: JIP/IEC/2018/0246<br><br>Informed written consent was obtained from study participants

Powered by Editorial Manager® and ProduXion Manager® from Aries Systems Corporation

**Format for specific study types**<br><br>**Human Subject Research (involving human participants and/or tissue)**<br>• Give the name of the institutional review board or ethics committee that approved the study<br>• Include the approval number and/or a statement indicating approval of this research<br>• Indicate the form of consent obtained (written/oral) or the reason that consent was not obtained (e.g. the data were analyzed anonymously)<br><br>**Animal Research (involving vertebrate animals, embryos or tissues)**<br>• Provide the name of the Institutional Animal Care and Use Committee (IACUC) or other relevant ethics board that reviewed the study protocol, and indicate whether they approved this research or granted a formal waiver of ethical approval<br>• Include an approval number if one was obtained<br>• If the study involved *non-human primates*, add *additional details* about animal welfare and steps taken to ameliorate suffering<br>• If anesthesia, euthanasia, or any kind of animal sacrifice is part of the study, include briefly which substances and/or methods were applied<br><br>**Field Research**<br><br>Include the following details if this study involves the collection of plant, animal, or other materials from a natural setting:<br>• Field permit number<br>• Name of the institution or relevant body that granted permission	
**Data Availability**<br><br>Authors are required to make all data underlying the findings described fully available, without restriction, and from the time of publication. PLOS allows rare exceptions to address legal and ethical concerns. See the PLOS Data Policy and FAQ for detailed information.	No - some restrictions will apply

A Data Availability Statement describing where the data can be found is required at submission. Your answers to this question constitute the Data Availability Statement and **will be published in the article**, if accepted.<br><br>**Important:** Stating 'data available on request from the author' is not sufficient. If your data are only available upon request, select 'No' for the first question and explain your exceptional situation in the text box.<br><br>Do the authors confirm that all data underlying the findings described in their manuscript are fully available without restriction?	
**Describe where the data may be found in full sentences. If you are copying our sample text, replace any instances of XXX with the appropriate details.**<br><br>• If the data are **held or will be held in a public repository**, include URLs, accession numbers or DOIs. If this information will only be available after acceptance, indicate this by ticking the box below. For example: *All XXX files are available from the XXX database (accession number(s) XXX, XXX.*).<br>• If the data are all contained **within the manuscript and/or Supporting Information files**, enter the following: *All relevant data are within the manuscript and its Supporting Information files.*<br>• If neither of these applies but you are able to provide **details of access elsewhere**, with or without limitations, please do so. For example:<br><br>*Data cannot be shared publicly because of [XXX]. Data are available from the XXX Institutional Data Access / Ethics Committee (contact via XXX) for researchers who meet the criteria for access to confidential data.*<br><br>*The data underlying the results presented in the study are available from (include the name of the third party*	Data cannot be shared publicly because this study has committed to protect the privacy of the data as per JIPMER Ethics Committee guidelines. However, data are available from the JIPMER Ethics Committee (contact via JIPMER Institutional Ethics Committee - Human Studies) for researchers who meet the criteria for access to confidential data.

and contact information or URL).<br>• This text is appropriate if the data are owned by a third party and authors do not have permission to share the data.<br><br>* typeset	
Additional data availability information:	

Powered by Editorial Manager® and ProduXion Manager® from Aries Systems Corporation

Covering letter

To
The Editor,

Sub: Submission of Manuscript for publication

Dear Editor,

I am submitting a manuscript titled **"Profile of risk factors for Non-Communicable Diseases (NCDs) in a highly urbanized district of India: Findings from district-wide STEPS Survey"**

In India, three out of the top five causes for morbidity and mortality are Non-Communicable Diseases (NCDs). Recent evidence from 'India-State level burden of diseases' indicated substantially higher prevalence of NCDs among affluent states of India compared to less developed ones. This called for state-specific estimates for NCDs and its determinants, especially in highly urbanized states, to deal effectively with the rising burden across Indian states. Puducherry is one of the highly urbanized states in the country which presents 'higher-middle epidemiological transition' indicating higher burden of NCDs. However, no systematic NCD risk factors survey had been conducted in the state.

We conducted this study in the district of Puducherry to estimate the prevalence of behavioral and biological risk factors of NCDs using the World Health Organization (WHO) prescribed STEPwise Surveillance (STEPS) methodology. We provided estimates of key NCDs and its risk factors based on gender, age group, and residence. The study showed that about one-third and one-fourth of men in the district were tobacco and alcohol users respectively. About half of the population was physically inactive and nine out of ten people were taking unhealthy diet. Generalized and abdominal obesity was present in half of the population, and hypertension and diabetes mellitus in one-third and one-fourth of the population respectively. High prevalence of various NCDs and its risk factors underscores the urgent need for population-based health promotion interventions with major emphasis on the urban population of the district.

The manuscript has not been submitted or accepted elsewhere. All the authors have contributed to, seen, and approved the final, submitted version of the manuscript. The authors declare no conflict of interest. We do not have any opposed reviewers for this manuscript.

I request you to please consider the manuscript for publication in your esteemed journal and look forward to a favorable decision.

Suggested editors:
1. Mahdieh Abbasalizad Farhangi, orcid.org/0000-0002-7036-6900, Tabriz University of Medical Sciences
2. Siddique A Abbasi, orcid.org/0000-0002-9601-7565, Brown University, UNITED STATES

Sincerely,

Dr Sitanshu Sekhar Kar

Additional Professor, Department of Preventive and Social Medicine

JIPMER, Puducherry - India

Manuscript

Click here to access/download;Manuscript;1. STEPS Manuscript (Plos submission).docx

Title Page

Profile of risk factors for Non-Communicable Diseases (NCDs) in a highly urbanized district of India: Findings from district-wide STEPS Survey

Short title: NCD Risk Factors profile in Puducherry district, India

Type: Original study

Authors: Parthibane Sivanantham1, Jayaprakash Sahoo2, Subitha Lakshminarayanan1, Zachariah Bobby3, Sitanshu Sekhar Kar1*

Author Affiliations:

[1]Department of Preventive and Social Medicine, Jawaharlal Institute of Postgraduate Medical Education and Research (JIPMER), Puducherry, India

[2]Department of Endocrinology, Jawaharlal Institute of Postgraduate Medical Education and Research (JIPMER), Puducherry, India

[3]Department of Biochemistry, Jawaharlal Institute of Postgraduate Medical Education and Research (JIPMER), Puducherry, India

Address for the corresponding author:

Dr. Sitanshu Sekhar Kar, Additional Professor, Department of Preventive and Social Medicine, JIPMER, Puducherry, **E-mail:** drsitanshukar@gmail.com

Abstract:

Introduction: Rapid urbanization and industrialization drives the rising burden of Non-Communicable Diseases (NCDs) worldwide that are characterized by uptake of unhealthy lifestyle such as tobacco and alcohol use, physical inactivity and unhealthy diet. In India, the prevalence of various NCDs and its risk factors shows wide variations across geographic regions necessitating region-specific evidence for population-based prevention and control of NCDs.

Objective: To estimate the prevalence of behavioral and biological risk factors of NCDs among adult population (18-69 years) in the district of Puducherry in South India.

Methodology: World Health Organization (WHO) prescribed STEPwise approach to NCD surveillance (STEPS) was conducted by following all three steps (behavioral, physical and biochemical measurements). A total of 2560 individuals were selected from urban and rural areas (50 clusters in each) through multi-stage cluster random sampling method. STEPS instrument was used to assess behavioral and physical measurements. Fasting blood sample was collected to estimate biochemical risk factors (Diabetes, Hypercholesterolemia) of NCDs.

Results: Among men, alcohol use 40.4% (95% CI: 37.4-43.4) was almost twice higher compared to tobacco use 24.4% (95% CI: 21.7-26.9). Nearly half of the population was physically inactive 45.8% (95% CI: 43.8-47.8) and obese 46.1% (95% CI: 44-48.1). Hypertension and diabetes mellitus was present among one-third 33.6% (95% CI: 31.6-35.5) and one-fourth 26.7% (95% CI: 24.1-29.1) of the population which were significantly higher among men. Physical inactivity and overweight/obesity increased with increasing education levels. Tobacco

and alcohol use was more common among men, whereas physical inactivity with obesity and hypercholesterolemia was higher among women.

Conclusion: The study highlights high prevalence of various NCDs and its risk factors among the adult population of Puducherry district. Population based health promotion interventions need to be initiated with major emphasis on the urban population of the district.

Key words:

Non Communicable Diseases, Risk factors, WHO STEPS, Hypertension, Diabetes, Obesity, INDIA

Introduction:

Non-Communicable Diseases (NCDs) are the leading cause for morbidity and mortality worldwide, with three-fourth of deaths occurring in the low and middle-income countries like India [1]. Between the years 1990 and 2016, disease burden in India due to NCDs increased from 48% to 75%. Notably, diabetes and ischemic heart diseases rose by 80% and 34% respectively. Currently, three out of the top five causes for morbidity and mortality in the country are NCDs [2].

Rising burden of NCDs is due to rapid urbanization and social development occurring in the country over the past two decades. The change occurring in social structures transforms lifestyles of populations that are mainly characterized by increased adaptation of unhealthy diet, physical inactivity and tobacco use [3]. These modifiable risk factors precede the development of metabolic risk factors and then progress to NCDs in populations [4]. This progression when not intervened with prompt population-based health promotion interventions, it causes a sizeable transition in the disease epidemiology of the population. Recent evidence from India-State level burden of diseases study revealed wide variations in epidemiological transition levels across Indian states indicating higher prevalence of NCDs among affluent states compared to less developed ones [2].

The geographical variation in NCD prevalence also informs the prevailing varying levels of NCD risk factors across the states. This warrants state-specific estimates for NCDs and its determinants, to deal effectively with the rising burden. Since 2010, with the launch of National Program for Prevention and Control of Cancer, Diabetes, CVD and Stroke (NPCDCS) and

adoption of National NCD Monitoring framework [5,6], several national and sub-national level surveys [7–10] have been conducted to estimate the prevalence of NCD risk factors across states in the country. However, except for tobacco, alcohol, overweight/obesity, and hypertension, state-specific estimates are currently unavailable for other NCD risk factors [11] which are crucial for devising population-specific interventions.

The state of Puducherry where this study was conducted is one of the highly urbanized states in the country [12]. Puducherry presents higher-middle epidemiological transition level which indicates higher burden of NCDs and its risk factors [2]. However, no systematic NCD risk factors survey has been conducted in the state. Therefore, this study was undertaken in the district of Puducherry to estimate the prevalence of behavioral and biological risk factors using the World Health Organization (WHO) prescribed STEPwise Surveillance (STEPS) methodology.

Methodology:

Study setting:

Puducherry is one of the four districts of the union territory of Puducherry located along the eastern coasts, in southern India. The district's population is 9.8 lakhs with majority (69.16%) residing in urban areas. The district has sex ratio of 1029, life expectancy (68.35 years), and literacy rate of 85.44% [13,14]. Puducherry stands seventh in Human Development Index (HDI) compared to other Indian states [15]. This survey was conducted in both urban and rural areas of Puducherry district.

Study design and sample size:

We conducted this NCD risk factors survey for a period of one year (February 2019 – February 2020). The sample size was obtained by following STEPS guidelines [4]. It was calculated using OpenEpi (version 3.01), considering 50% prevalence of risk factors, 95% confidence interval and design effect of 1.5. The sample size of 2304 was obtained after summating across gender and residence. This was sufficient to provide districtwide representative results across gender and residence. Assuming response rate of 90%, sample size was raised to 2560 which was proportionately divided between urban (n=1770) and rural (n=790) areas as per respective population distribution (69.16% vs 30.84%).

Sampling method:

Multistage, stratified, geographically clustered sampling method was employed using Census 2011 data as the baseline. Three and two-staged sampling was done in urban and rural areas respectively. Of all urban wards (90 in total) and revenue villages (62 in total) in the district, fifty clusters each, from urban and rural areas were chosen (100 clusters in total) in the first sampling stage. In urban areas, one Census Enumeration Block (CEB) was chosen randomly from each selected ward in the second stage. From each selected CEB and village, 36 and 16 households were chosen respectively using systematic random sampling in the final sampling stage. In each selected household, one adult (18-69 years) member resided in the household for at least past six months was chosen using KISH method [16].

Data collection instrument:

WHO STEPS (version 3.2) questionnaire was culturally adapted, translated to Tamil, and pilot tested before data collection. Risk factors were assessed in three steps. Behavioral risk factors such as tobacco use, alcohol consumption, physical inactivity, inadequate fruits and vegetables intake, were assessed in step one. Physical measurements for body mass index (BMI) and abdominal obesity were done in step two. Biochemical risk factors (fasting blood glucose and total cholesterol) were assessed in third step by collecting blood samples from alternate participants.

Data collection procedure:

In each CEB or village, a household was chosen randomly using respective map of the cluster. Subsequently, the required number of households in the cluster was selected. Data were collected from an eligible household member by a trained investigator after obtaining informed written consent. All three survey steps were conducted at households. When household members were unavailable, two more visits were made at times convenient to them. Those couldn't be contacted after two visits, were considered as non-response. The study was permitted by the Institute's Ethics Committee. *(JIP/IEC/2018/0246)*

Behavioral risk factors (Step 1):

Demographic characteristics such as age, gender, residence, education, occupation including contact details were collected initially. Current tobacco (smoke and smokeless forms) and alcohol (quantity) use, and intake of fruits and vegetables (quantity and frequency) were

collected. Physical activity at work, travel and leisure activities were assessed using Global Physical Activity Questionnaire (GPAQ). Showcards were used to describe physical activity (type and intensity), standard drink (alcohol) and a serving of fruits and vegetables.

Physical measurements (Step 2):

Height, weight, waist circumference (WC) and blood pressure (BP) were measured by following standard guidelines. Participant's height was measured in standing posture with barefoot and light clothing. Measurements were done using portable stadiometer and electronic weighing scale at nearest 0.1cm and 100gm respectively. SECA constant tape was used for waist measurement. Three BP readings, each at three minutes apart were captured using electronic device *(OMRON, HEM 7120, Omron Corporation, Kyoto, JAPAN),* and the last two readings were averaged to determine the BP status. Pregnant women were not subjected to physical measurements. All equipments were calibrated regularly before and during data collection.

Biochemical measurements (Step 3):

Fasting blood samples were collected from alternate participants (50% of samples). Each consenting participant of step 3 was given a handout describing the need for overnight fasting (10-12 hours). Blood sample was collected in seated position on the next day morning, and was transported to the institute's biochemistry laboratory for estimation.

Fasting blood glucose and total cholesterol were estimated using commercially available kits adapted to clinical chemistry autoanalyzer based on spectrophotometry *(Beckman Coulter Inc,*

Brea, California, USA). Glucose oxidase peroxide and cholesterol oxidase peroxide were used for the estimation at 520nm.

All information was collected in electronic version of the questionnaire loaded in the field data collection software ODK Collect (version 1.25.1).

Operational definitions:

Behavioral risk factors were determined based on the cut-offs recommended by STEPS guidelines [4]. Tobacco (smoke or smokeless) and alcohol use in last thirty days and one year respectively, was considered as current use. Alcohol use was assessed as Standard drinks (one standard drink = 100ml wine or 285ml bear or 30ml Spirit / Toddy / Arrack). Men and women consumed at least six and four standard drinks respectively, in at least one occasion during last 30 days, were considered to have harmful use of alcohol. Along with type of physical activity (work, travel and leisure), intensity levels (low, moderate and severe) were also captured. This was factored with the duration of such physical activities to derive metabolic equivalent time (MET-minutes/week). Those had less than 600 MET, 600-1500 MET, and more than 1500 were classified to have low, moderate and high levels of physical activity. Behavioural assessment was based on self-report.

Overweight (23 to 24.9kg/m^2) and obesity (≥25kg/ kg/m^2) were determined by following Asian cut-offs of BMI classification. WC ≥90cm for men and ≥80cm for women were regarded as abdominal obesity [17]. Systolic BP (SBP) of ≥140mm Hg or Diastolic BP (DBP) of ≥90mm Hg or currently on hypertension lowering drugs was considered as raised BP (hypertension) [18].

Diabetes mellitus (DM) was determined at Fasting plasma glucose (FPG) of ≥126 mg/dl or currently on anti-diabetic medications [19]. Hypercholesterolemia was defined as those having total cholesterol level of ≥200mg/dl [19] or currently on lipid-lowering drugs.

Statistical analysis:

Data cleaning and analysis were conducted using STATA (StataCorp LP, College Station, TX, USA). Continuous variables such as age, quantity of tobacco sticks and alcohol use, and categorical variables such as prevalence of various risk factors (tobacco and alcohol use, physical inactivity, raised BP, diabetes etc.) were summarized using mean (SD) and proportions respectively. All estimates were presented with 95% CI. Significant differences in risk factors prevalence between sub-groups of independent variables were determined by comparing 95% CI.

The effect of clusters (villages/wards) on dependent variables was assessed using Intraclass Correlation Coefficient (ICC) and presence of statistically significant difference in the intercept only mixed-effects model compared to standard binary logistic regression. To those dependent variables showed statistically significant improvement in the model fit, forward weighted adjusted prevalence ratio were determined using 'multilevel mixed-effects generalized linear model' under 'Poisson's regression'. Those variables showed non-significant improvement in model fit, weighted forward stepwise generalized linear modelling using 'Poisson's regression' was done to determine the prevalence ratio. The models were developed for each risk factor by keeping the risk factor as dependent variable and socio-demographic characteristics (gender,

age group, education, marital status, occupation) as independent variables. In regression models, p≤0.05 were considered statistically significant.

Results:

The response rate for the steps 1&2 and 3 were 2415/2560 (94.3%) and 1117/1208 (92%) respectively. Among the participants, mean (SD) age was 44.3 [14] years, majority belonged to urban area (69.6%), women 1329/2560 (55%), and nearly half received at least secondary education 1173/2415 (48.6%) and also employed 1237/2415 (51.2%). Socio-demographic characteristics are provided in the table 1.

Table 1: Socio-demographic profile of study participants (N= 2415)

Variables	Male (n=1086) n (%)	Female (n=1329) n (%)	Both Gender (N=2415) n (%)
**Age categories (in years)**			
18-44	538 (49.5)	667 (50.2)	1205 (49.9)
45-69	548 (50.5)	662 (49.8)	1210 (50.1)
**Residence**			
Urban	749 (69)	932 (70.1)	1681 (69.6)
Rural	337 (31)	397 (29.9)	734 (30.4)
**Educational status**			
No formal education	67 (6.2)	223 (16.8)	290 (12)
Less than primary	121 (11.1)	193 (14.5)	314 (13)
Primary education	287 (26.4)	348 (26.2)	635 (26.3)
Secondary/ Higher Secondary	374 (34.4)	360 (27.1)	734 (30.4)
Graduation and above	235 (21.6)	204 (15.3)	439 (18.2)
Refused to answer	2 (0.2)	1 (0.1)	3 (0.1)
**Religion**			
Hindu	1027 (94.6)	1243 (93.5)	2270 (94)
Christian	38 (3.5)	56 (4.2)	94 (3.9)
Muslim	21 (1.9)	28 (2.1)	49 (2)
Others	0	2 (0.2)	2 (0.1)
**Marital status**			
Never married	228 (21)	90 (6.8)	318 (13.2)

Currently married	831 (76.5)	988 (74.3)	1819 (75.3)
Divorced/Separated	6 (0.6)	20 (1.5)	26 (1.1)
Widowed	21 (1.9)	231 (17.4)	252 (10.4)
**Occupation**			
Government employee	42 (3.9)	19 (1.4)	61 (2.5)
Non-Government employee	40.6 (37.4)	184 (13.8)	590 (24.4)
Self employed	403 (37.1)	183 (13.8)	586 (24.3)
Student	56 (5.2)	29 (2.2)	85 (3.5)
Homemaker	10 (0.9)	806 (60.6)	816 (33.8)
Retired	57 (5.2)	14 (1.1)	71 (2.9)
Unemployed (able to work)	49 (4.5)	34 (2.6)	83 (3.4)
Unemployed (Unable to work)	63 (5.8)	60 (4.5)	123 (5.1)

The mean (CI) of various behavioral risk factors and physical measurements are given in table 2. Fruits and vegetables intake and physical activity were significantly higher among men. The intake levels were significantly higher among urban population, whereas physical activity was significantly higher among rural population. BMI was significantly higher among women 25.8% (95% CI: 25.5-26). Fasting blood glucose was higher among men, while total cholesterol was higher among women 183 (95% CI: 180-186) and urban population 184 (95% CI: 181-187).

Table 2: Mean (Confidence Interval) of various behavioral and physical measurements and biochemical risk factors by age group, gender and residence among the study participants (N= 2415)

**Variables**	**Mean (CI) of behavioral risk factors on an average per day**			
	**Daily tobacco use (smoke form)**	**Standard drinks of alcohol consumed**	**Servings of fruits and vegetables intake**	**MET of physical activity/week**
**Age (in years)**				
18-44	6.65 (5.47-7.83)	4.97 (4.5-5.4)	3.1 (3.0-3.2)	1697 (1532-1862)
45-69	11.64* (9.6-13.7)	6.33* (5.7-6.9)	2.8* (2.7-2.9)	1762 (1600-1925)
**Gender**				
Male	9.55* (8.2-10.9)	5.66 (5.3-6.0)	3.1 (3.0-3.2)	2068 (1879-2257)
Female	-	3.25 (0-6.53)	2.8* (2.7-2.9)	1454* (1313-1594)
**Residence**				
Urban	10.2 (8.5-12.0)	5.7 (5.3-6.2.0)	3.2 (3.1-3.3)	1371* (1239-1504)
Rural	7.9 (6.0-10)	5.5 (4.8-6.2.0)	2.5* (2.4-2.7)	2551 (2333-2769)
**Overall**	9.58 (8.24-10.92)	5.63 (5.25-6.0)	2.94 (2.9-3.0)	1730 (1614-1845)

	BMI	Waist Circumference	Systolic Blood Pressure	Diastolic Blood pressure
**Age (in years)**				
18-44	25 (24.7-25.5)	90.6 (89.9-91.2)	120 (119-121)	79.8* (79.0-80.4)
45-69	25 (24.9-25.0)	91 (90.0-91.7)	128* (127-129)	82.9 (82.0-83.5)
**Gender**				
Male	24.3 (23.9-24.7)	88.6 (88.0-89.0)	127* (126-128)	83.6* (83.0-84.3)
Female	25.8* (25.5-26.0)	92.6* (91.9-93.3)	122 (121-123)	79.5 (79.0-80.0)
**Residence**				
Urban	25.5 (25.0-26.0)	92* (91.5-92.5)	125* (124-126)	82* (81.4-82.5)
Rural	24.3 (23.9-34.6)	87 (86.0-88.0)	122 (121-123)	80 (79.0-81.0)
**Overall**	25.1 (24.9-25.4)	90.8 (90.0-91.3)	124.3 (123.6-125)	81.4 (81.0-81.8)

	**Mean (Confidence Interval) of biochemical risk factors**	
	**Fasting Blood Glucose**	**Total Cholesterol**
**Age (in years)**		
18-44	105 (101-110)	174 (171-177)
45-69	119* (115-124)	188* (184-191)
**Gender**		
Male	118 (112-123)	179 (175-183)
Female	110 (105-113)	183 (180-186)
**Residence**		
Urban	116 (112-120)	184* (181-187)
Rural	106 (100-112)	175 (171-180)
**Overall**	113 (110-116)	182 (179-184)

*Indicates statistically significant difference between the sub-groups

The prevalence of current tobacco and alcohol use were 12.3% (95% CI: 11-13.7) and 18.5% (95% CI: 16.9-20) respectively, which were significantly higher among men. Alcohol use 40.6% (95% CI: 37.5-43.9) was almost twice higher compared to tobacco use 24.4% (95% CI: 21.7-26.9) among men. About half 45.8% (95% CI: 43.8-47.8) of the population was physically inactive and it was nearly twice higher among urban residents. Nearly nine out of ten people 86.8% (95% CI: 85.4-88.1) in Puducherry consumed fruits and vegetables in inadequate amounts. (table 3)

Prevalence of obesity 46.1% (95% CI: 44-48.1) was more than twice higher in the study population when compared to overweight 17.6% (95% CI: 16.1-19.1). One-third 33.6% (95% CI: 31.6-35.5) of the population had hypertension that was significantly higher among men and urban residents. DM was present in one-fourth 26.7% (95% CI: 24.1-29.1) of the population, with significantly higher prevalence among men. Over one-third 34.8% (32.5-37.5) had hypercholesterolemia that was significantly higher among urban population. (table 3)

Table 3: Prevalence of various risk factors of NCDs by age group, gender and residence among the study participants:

Variables	Current tobacco use, % (CI)	Current use of alcohol, % (CI)	Harmful use of alcohol, % (CI)
**Age (in years)**			
18-44	11.5 (9.9-13.4)	19.1 (16.7-21.3)	8.8 (7.2-10.5)
45-69	13 (11.1-15)	17.9 (15.7-20)	10.3 (8.6-12)
**Gender**			
Male	24.4* (21.7-26.9)	40.6* (37.5-43.9)	21* (18.7-23.5)
Female	2.3 (1.6-3.2)	0.4 (0.1-0.8)	0.2 (0-0.5)
**Residence**			
Urban	12.7 (11.1-14.4)	18.1 (16.4-20.1)	9.5 (8-10.9)
Rural	11.3 (9.1-13.8)	19.2 (16.1-22.1)	9.7 (7.5-11.7)
**Overall**	12.3 (11-13.7)	18.5 (16.9-20)	9.6 (8.4-10.8)
	**Low physical activity, % (CI)**	**Inadequate intake of fruits and vegetables, % (CI)**	**Raised Blood pressure, % (CI)**
**Age (in years)**			
18-44	48.6 (45.6-51.5)	83.7 (81.7-85.8)	20.7 (18.7-23.3)
45-69	42.9 (40.1-45.6)	89.8 (88.1-91.5)	46.5* (43.7-49.5)
**Gender**			
Male	41.5 (38.5-44.5)	83.4 (81.1-85.6)	37.1* (34.3-40.1)
Female	49.2* (46.6-51.8)	89.5 (87.9-91)	30.8 (28.4-33.2)
**Residence**			
Urban	52.9* (50.7-55.5)	85.5 (83.8-87.2)	36* (33.8-38.5)
Rural	29.3 (26-32.6)	89.8* (87.6-91.8)	28.2 (25.1-31.3)
**Overall**	45.8 (43.8-47.8)	86.8 (85.4-88.1)	33.6 (31.6-35.5)
	**Overweight, % (CI)**	**Obesity, % (CI)**	**Abdominal obesity, % (CI)**
**Age (in years)**			
18-44	16.8 (14.6-19)	45.1 (42.4-48)	67.4 (64.8-70)
45-69	18.3 (16.4-20.6)	47 (44.2-49.9)	69.1 (66.4-71.7)

**Gender**			
Male	21.7* (19.4-24.2)	37.5 (34.7-40.2)	46 (43.1-49.2)
Female	14.2 (12.4-15.9)	53.1* (50.4-56)	86.5* (84.6-88.3)
**Residence**			
Urban	18.5 (16.6-20.3)	49.2* (46.8-51.6)	71.9* (69.7-74)
Rural	15.6 (13-18.3)	38.9 (35.3-42.4)	59.8 (56.2-63.6)
**Overall**	17.6 (16.1-19.1)	46.1 (44-48.1)	68.2 (66.4-70.1)

	**Diabetes Mellitus, % (CI)**	**Hypercholesterolemia, % (CI)**
**Age (in years)**		
18-44	16.5 (13.4-19.7)	24.4 (20.9-27.9)
45-69	35.3* (31.8-38.9)	43.7* (39.4-47.6)
**Gender**		
Male	31.6* (27.5-35.7)	34.6 (30.5-39.3)
Female	23.2 (20.1-26.6)	35 (31.6-38.6)
**Residence**		
Urban	27.6 (24.5-30.9)	37.9* (34.7-41.1)
Rural	24.4 (20.1-29.1)	27.3 (22.7-32.0)
**Overall**	26.7 (24.1-29.1)	34.8 (32.5-37.5)

*Indicates statistically significant difference between the sub-groups

Socio-demographic determinants of various risk factors are given in table 4. The probabilities of tobacco and alcohol use were significantly higher among men by 26% (PR: 1.26, 95% CI: 1.23-1.3) and 49% (PR: 1.49, 95% CI: 1.4-1.54) respectively. Both these risk factors declined with increasing levels of education. Physical inactivity was significantly higher among urban population (PR: 1.24, 95% CI: 1.17-1.31), while showing gradual increase in its prevalence with increasing levels of education. Hypertension (PR: 0.9, 95% CI: 0.86-0.95) and DM (PR: 0.9, 95% CI: 0.84-0.96) were significantly lower among women by about 9%. Overweight and obesity also increased steadily across increasing education levels.

Table 4: Determinants of various risk factors of NCDs in the population of Puducherry district (N=2415)

Variables	Current tobacco use PR (95% CI)	Current alcohol use PR (95% CI)	Physical Inactivity PR (95% CI)	Insufficient Servings PR (95% CI)	Raised Blood Pressure PR (95% CI)	Raised Blood Glucose PR (95% CI)	Overweight /obesity PR (95% CI)	Raised Cholesterol PR (95% CI)
**Gender**								
Male	1.26* (1.23-1.3)	1.49* (1.4-1.54)	1	1	1	1	1	1
Female	1	1	1.04 (0.98-1.09)	1.06* (1.03-1.1)	0.9* (0.86-0.95)	0.9* (0.84-0.96)	1.02 (0.97-1.08)	1.04 (0.95-1.13)
**Residence**								
Rural	1	1	1	1	1	1	1	1
Urban	1.03 (0.99-1.06)	1.01 (0.98-1.04)	1.24* (1.17-1.31)	0.96 (0.92-1)	1.09* (1.05-1.14)	1.03 (0.97-1.09)	1.12* (1.07-1.17)	1.1* (1.03-1.2)
**Age (in years)**								
18-44	1	1	1	1	1	1	1	1
45-69	0.97 (0.94-1)	0.95* (0.92-0.98)	0.99 (0.93-1.04)	1.04 (1-1.08)	1.22* (1.16-1.27)	1.13* (1.06-1.2)	1.03 (0.99-1.08)	1.19* (1.11-1.27)
**Education**								
No formal education	1	1	1	1	1	1	1	1
Primary education	0.96 (0.92-1)	0.99 (0.96-1.03)	1.09* (1.01-1.17)	1.01 (0.9-1.04)	1.04 (0.97-1.11)	1.04 (0.95-1.13)	1.1* (1.02-1.18)	1.03 (0.94-1.14)
Secondary education	0.92* (0.88-0.97)	0.93* (0.88-0.96)	1.14* (1.05-1.23)	0.99 (0.95-1.03)	0.96 (0.9-1.04)	1.01 (0.9-1.1)	1.13* (1.05-1.23)	1 (0.9-1.13)
Graduation and above	0.87* (0.82-0.93)	0.89* (0.84-0.94)	1.25* (1.14-1.37)	0.97 (0.92-1.03)	0.97 (0.9-1.05)	0.98 (0.88-1.09)	1.2* (1.09-1.3)	0.96 (0.85-1.09)
**Marital status**								
Currently married	1	1	1	1	1	1	1	1
Never married	1.03 (0.98-1.09)	1.02 (0.95-1.08)	0.94 (0.88-1.01)	0.97 (0.92-1)	0.89* (0.84-0.94)	0.85* (0.78-0.91)	0.8* (0.75-0.86)	0.97 (0.88-1.07)
Widowed/divorced	1.07* (1.02-1.12)	0.99 (0.96-1.02)	1 (0.92-1.09)	1 (0.96-1.04)	1.06 (0.9-1.14)	1.06 (0.97-1.16)	1 (0.93-1.07)	0.99 (0.9-1.1)
**Occupation**								
Home maker	1	1	1	1	1	1	1	1
Government employee	0.98 (0.92-1.05)	0.98 (0.89-1.09)	0.85* (0.73-0.98)	0.94 (0.84-1.06)	1.04 (0.91-1.18)	1.1 (0.94-1.3)	1.04 (0.93-1.17)	1.01 (0.8-1.2)
Non-government employee	1.01 (0.98-1.04)	1.02 (0.98-1.05)	0.93* (0.86-0.99)	1.04* (1.01-1.08)	0.99 (0.93-1.04)	0.99 (0.92-1.07)	0.93* (0.87-0.98)	1.09 (1-1.19)
Self-employed	1.03 (0.99-1)	1.05* (1.02-1.09)	0.91* (0.85-0.97)	1.01 (0.97-1.04)	0.97 (0.91-1.03)	0.95 (0.88-1.03)	0.93* (0.88-0.99)	1.05 (0.96-1.14)
others	0.96 (0.92-1)	0.93* (0.89-0.97)	0.95 (0.9-1)	1.03 (0.98-1.08)	1.01 (0.94-1.07)	1.12* (1.02-1.23)	0.86* (0.81-0.93)	1 (0.92-1.1)

*Significant results: p≤0.05, PR: Prevalence Ratio, CI: Confidence Interval

Discussion:

We conducted this survey to estimate the prevalence of various key risk factors of NCDs using WHO prescribed STEPS methodology. Socio-demographic predictors of the risk factors were also determined.

The study revealed a marginal increase (12.3%) in tobacco use in Puducherry district in comparison to the Global Adult Tobacco Survey (GATS-2) finding (11.2%) for the district [20]. The increase in tobacco use was much pronounced among males (17.7% in GATS to 24.4%), and in urban areas (7.5% in GATS to 12.7%). On the other hand, tobacco use among women has shown a considerable decline (5.1% in GATS to 2.3%) [20]. The surge in smoked tobacco in the district raises concerns, as Puducherry has shown a substantial decline in tobacco use during the last decade (GATS 1 vs 2) [20,21]. Recent program initiatives such as formation of district-level coordination committee and field task force shall provide the necessary impetus for better planning, coordination, implementation and monitoring the Cigarettes and Other Tobacco Products Act, 2003 (COTPA) violations, which might drive down the rising prevalence signaled from this study.

Over one-third (40.4%) of men in Puducherry district were current alcohol user. This was comparable to the National Family Health Survey-4 (NFHS) prevalence (41%) for the district, [22] but higher than the national prevalence (29.2%) [23]. The observed prevalence was less compared to the neighbouring state of Tamilnadu (46.7%), but higher than Kerala (37%) and Karnataka (29.3%) [24]. Studies carried out using STEPS methodology in other Indian states showed prevalence between 31.1% and 40%. (25–27) NFHS-4 was done among the 15-45 years

age group and only a minority were men (14%) which tend to underestimate the alcohol prevalence for the district [24]. Considering this, the alcohol prevalence observed in the study that is comparable to NFHS-4 finding indicates the rising levels of alcohol use in the district. This carries an important public health implication, as it has been well established that South Asians taking more than moderate level of alcohol have increased risk for Coronary Artery Diseases [28].

Nearly half (45.8%) of study population was physically inactive and it was significantly higher among women (49.2%) and urban areas (52.9%). This prevalence was much lesser when compared to Tamilnadu (65.8%), a neighbouring state having the population socio-demographic characteristics same as this study [7]. Similar to the current finding, a recent study from Puducherry also reported physically inactivity among half (49.7%) of urban population [29]. Another study conducted across seven states in India found differential levels of physical inactivity ranging between 42.3% and 81.2%. These findings although evolved from varied regions, they clearly implied a markedly higher prevalence among both men (38.8%) and women (46.1%) in India [7]. The wide-ranging prevalence also signifies the need for state-specific determinants in order to implement evidence-based population-specific interventions to promote physical activity in the country. Considering that physical inactivity is significantly higher among urban population, and increasing urbanization of rural areas in the years ahead [30], future studies needs to examine the extent of pedestrian-friendly neighbourhoods, public access to parks, open spaces, public transport and other social amenities across states, which are proven to improve physical activities at population level [31].

Substantiating the higher levels of physical inactivity, two-third (63.7%) of the study population was overweight or obese. This was twice higher compared to NFHS-4 finding (36%) for the district [22]. The urban prevalence (67.7%) of overweight or obesity was also higher as compared to a more recent survey (64.4%) from Puducherry [32]. The markedly increased prevalence of this risk factor calls for immediate population-level obesity prevention and control programs to target women, urban residents and school children of Puducherry district. This public health action gains importance because evolving evidences predict doubling of overweight, and tripling of obesity levels in the country by 2040 [33] which are mainly linked to sedentary and changing dietary habits across populations.

In India, dietary risk factor is the leading cause for morbidity and mortality among the states experiencing high epidemiological transition like Puducherry [2]. In congruence, nearly nine out of 10 people (86.8%) in Puducherry district consumed less than recommended amount of fruits and vegetables. A survey from urban Puducherry also reported much higher prevalence (97.5%) as compared to the current study finding (85.5%) [32]. India, despite being the second-largest producer of fruits and vegetables [34], low intake of fruits and vegetables is highly prevalent across Indian states ranging from 76% in Maharashtra to 99% in Tamilnadu [7]. Similar higher prevalence was also reported by NFHS-3, while showing decreasing levels of intake with decreasing economic status [35]. Addressing this situation requires multi-sectoral, multi-level and multi-pronged interventions to promote indigenous fruits and vegetables, and streamline its supply chain, which are crucial to enhance 'availability and affordability' of fruits and vegetables across Indian states.

One-third of population in Puducherry district had hypertension (33.6%) that was significantly higher among men (37.1%) and urban population (36%). This finding was almost three times higher when compared to NFHS-4 finding for the district (12.25%) [22], and higher than national (29.8%) and pooled prevalence for south India (26.4%) [36]. The urban prevalence (36%) was also higher compared to a recent study (32.5%) from urban Puducherry [32]. The large discordance in the prevalence, in comparison to NFHS-4 shall be attributed to relatively younger population (15-45 years) and over-representation of women (86%) in the later survey [24]. Significantly higher prevalence ratio among men and 45-69 years age group in the current study also substantiates the observed discordance with NFHS-4 finding. Higher prevalence among men and urban population were also reported in other Indian studies [10,37].

Diabetes is the leading metabolic risk factor in claiming highest disability adjusted life years (DALYs), in those Indian states experiencing high epidemiological transition [38] like Puducherry. Prevalence of diabetes in the study (26.7%) was substantially higher than the national prevalence (7.3%) [8]. Its prevalence was also higher compared to socio-economically comparable India states like Tamilnadu (17.5%), Kerala, (14.8%) Punjab (15.5%). The higher prevalence reported in the current study needs to be interpreted in relation to the time points when those compared studies were conducted. Because the prevalence of diabetes in India has increased by 1.78 times over the last decade across Indian states which might have corresponded to higher prevalence in the current study [39]. Further, a large scale study from Tamilnadu showed that during the decade from 2006 to 2016 the prevalence of diabetes had increased marginally in cities by 8%, but significantly in towns (39%) and villages (34%) [40].

This also substantiates the higher prevalence observed, especially in the rural population of Puducherry (24.4%) which was comparable to several urban populations of the country.

Hypercholesterolemia is one of the key modifiable risk factors for cardiovascular diseases. Its prevalence in Puducherry district (34.8%) was significantly higher compared to the prevalence from four Indian states (13.9%) that ranged between 4.3% (Jharkhand) and 18.3% (Tamilnadu) [41]. The current study finding was also higher compared to a recent study from Tamilnadu (31.4%) [42]. The difference observed between the four states and current study could be due to the methodological differences, as the former survey assessed cholesterol levels among every fifth participant [41]. However, the regional disparity presented across states reaffirms the need for state-specific estimates and determinants in the country which are crucial for devising community-based targeted interventions such as screening, dietary advice, improving physical activity across populations.

The prevalence of various NCDs and its risk factors in the study population was alarming, yet not unanticipated, given the established findings from the states experiencing similar epidemiological transition, as Puducherry [25,27,40,42,43]. Recent evidences also presents a positive correlation between urbanization and prevalence of NCD risk factors such as physical inactivity, raised BMI, and low intake of fruits and vegetables [44]. In cognizant of the rising burden, several key initiatives have been undertaken in the country especially in the current decade. India became the first country to adopt the Global NCD Monitoring Framework to set a target of 25% relative reduction in premature NCDs deaths by 2025 [5]. In line with this, National Multi-sectoral action plan has been developed to drive down various NCD risk factors,

recognizing the required interventions from departments beyond health [45]. Importantly, comprehensive primary care, population-based health promotion interventions, and financial protection being central to any state in the country for effective NCD prevention and control, is delivered through 'Ayushman Bharat' that was launched in 2018 [46]. However, the impact of these newer interventions and the ongoing ones delivered through NPCDCS need to be observed in the years to come.

Limitations:

Interview based nature of the study could have underestimated a few behavioral risk factors due to socially desirable responses. Behavioral risk factors assessed depended on participant's ability to recall their health behaviors which might have led to recall bias to some extent. However, interviews conducted by trained investigator and use of standardized STEPS questionnaire and show cards to facilitate recall of health risk behaviors helped to minimize these biases. In the study, physical inactivity levels were determined using GPAQ questionnaire - a globally accepted scale. But, emerging evidences raises concerns over underestimation of household and leisure kind of physical activities if GPAQ is used [47,48]. This possibly explains the higher physical inactivity levels observed among women in the study.

Conclusion:

The study highlights higher prevalence of various behavioral and biological risk factors of NCDs including its determinants for the district of Puducherry. Notably, one third and one-fourth men in the district were tobacco and alcohol users respectively. About half of the population was

physically inactive (45.8%) and nine out of ten people (86.8%) were taking unhealthy diet. These behavioral risks corresponded to higher levels of metabolic risk factors by showing generalized (46.1%) and abdominal obesity (68.2%) in half of the population and hypertension and diabetes mellitus in one-third (33.6%) and one-fourth (26.7%) of the population respectively. In the population, increasing education levels decreased tobacco and alcohol but raised physical inactivity and obesity. The higher prevalence of these risk factors, beyond serving as the baseline estimates for NCD risk factors in the district, underscores the urgent need for population based health promotion interventions which are indispensable for rendering effective prevention and control of NCDs in the district of Puducherry.

References:

1. World Health Organization Regional Office for the Western. Global status report on noncommunicable diseases 2010 [Internet]. World Health Organization; Report No.: ISBN 978 92 4 068645 8. Available from: https://www.who.int/nmh/publications/ncd_report_full_en.pdf

2. Indian Council of Medical Research, Public Health Foundation of India, Institute for Health Metrics And Evaluation. India: Health of the Nation's States (India State-Level Disease Burden Initiative) [Internet]. [cited 2020 May 9]. Report No.: ISBN 978-0-9976462-1-4. Available from: https://phfi.org/the-work/research/the-india-state-level-disease-burden-initiative/

3. Reddy KS. Cardiovascular diseases in the developing countries: dimensions, determinants, dynamics and directions for public health action. Public Health Nutr. 2002 Feb;5(1A):231–7.

4. World Health Organization. Non Communicable Diseases (STEPwise Approach to Non Communicable Diseases - STEPS Manual) [Internet]. WHO. [cited 2017 Dec 10]. Available from: http://www.who.int/ncds/surveillance/steps/en/

5. National Stroke Registry Program - Fact Sheet - NCD Monitoring Framework [Internet]. [cited 2020 Apr 20]. Available from: http://ncdirindia.org/stroke/BS_FactSheet_MonitoringFramework.aspx

6. National Programme for Prevention and Control of Cancer, Diabetes, Cardiovascular diseases and Stroke (NPCDCS) | Ministry of Health and Family Welfare | GOI [Internet]. [cited 2020 Jan 9]. Available from: https://mohfw.gov.in/Major-Programmes/non-communicable-diseases-injury-trauma/Non-Communicable-Disease-II/National-Programme-for-Prevention-and-Control-of-Cancer-Diabetes-Cardiovascular-diseases-and-Stroke-NPCDCS

7. National Institute of Medical Statistics and Division of Non-Communicable Diseases, Indian, Council of Medical Research, New Delhi. Integrated Disease Surveillance Project (IDSP) Non-Communicable Disease Risk Factors Survey [Internet]. 2007 [cited 2018 May 29]. Available from: http://www.who.int/ncds/surveillance/steps/2007_STEPS_Report_India_7States.pdf

8. Anjana RM, Deepa M, Pradeepa R, Mahanta J, Narain K, Das HK, et al. Prevalence of diabetes and prediabetes in 15 states of India: results from the ICMR-INDIAB population-based cross-sectional study. Lancet Diabetes Endocrinol. 2017;5(8):585–96.

9. Tripathy JP, Thakur JS, Jeet G, Chawla S, Jain S, Prasad R. Urban rural differences in diet, physical activity and obesity in India: are we witnessing the great Indian equalisation? Results from a cross-sectional STEPS survey. BMC Public Health [Internet]. 2016 Aug 18 [cited 2020 Mar 13];16. Available from: https://www.ncbi.nlm.nih.gov/pmc/articles/PMC4989330/

10. Thakur JS, Jeet G, Nangia R, Singh D, Grover S, Lyngdoh T, et al. Non-communicable diseases risk factors and their determinants: A cross-sectional state-wide STEPS survey, Haryana, North India. PLOS ONE. 2019 Nov 27;14(11):e0208872.

11. Nethan S, Sinha D, Mehrotra R. Non Communicable Disease Risk Factors and their Trends in India. Asian Pac J Cancer Prev APJCP. 2017 27;18(7):2005–10.

12. Level of Urbanisation : Ministry of Housing and Urban Affairs, Goverment of India [Internet]. [cited 2020 May 26]. Available from: http://mohua.gov.in/cms/level-of-urbanisation.php

13. Puducherry (Pondicherry) District Population Census 2011-2020, Puducherry literacy sex ratio and density [Internet]. [cited 2020 May 26]. Available from: https://www.census2011.co.in/census/district/482-puducherry.html

14. Sarala G. A Situational Analysis of Women and Girls in Pondicherry [Internet]. New Delhi: National Commission for Women; Available from: http://ncwapps.nic.in/pdfReports/Gender_Profile_Pondicherry.pdf

15. Sub-national HDI - Subnational HDI - Global Data Lab [Internet]. [cited 2020 Apr 6]. Available from: https://globaldatalab.org/shdi/shdi/IND/?interpolation=0&extrapolation=0&nearest_real=0&years=2018

16. Kish L. A Procedure for Objective Respondent Selection within the Household. J Am Stat Assoc. 1949 Sep 1;44(247):380–7.

17. WHO Expert Consultation. Appropriate body-mass index for Asian populations and its implications for policy and intervention strategies. Lancet Lond Engl. 2004 Jan 10;363(9403):157–63.

18. James PA, Oparil S, Carter BL, Cushman WC, Dennison-Himmelfarb C, Handler J, et al. 2014 Evidence-Based Guideline for the Management of High Blood Pressure in Adults: Report From the Panel Members Appointed to the Eighth Joint National Committee (JNC 8). JAMA. 2014 Feb 5;311(5):507–20.

19. Association AD. Standards of Medical Care in Diabetes—2016 Abridged for Primary Care Providers. Clin Diabetes. 2016 Jan 1;34(1):3–21.

20. World Health Organization, Ministry of Health and Family Welfare, TATA Institute of Social Sciences (TISS). Global Adult Tobacco Survey, Fact Sheet, India (2016-2017) [Internet]. [cited 2020 Apr 5]. Available from: https://tiss.edu/uploads/files/32_PD.pdf

21. Tata Institute of Social Sciences (TISS), Ministry of Health and Family Welfare. Global Adult Tobacco Survey GATS 1 India 2009-10 [Internet]. [cited 2019 Apr 18]. Available from: http://www.searo.who.int/tobacco/documents/2010-pub2.pdf

22. Indian Institute of Population Sciences (IIPS), Ministry of Health and Family Welfare. National Family Health Survey 4 (2015-16): State Fact Sheet [Internet]. [cited 2020 Jun 5] p. 1–6. Available from: http://rchiips.org/nfhs/pdf/NFHS4/PY_FactSheet.pdf

23. Indian Institute of Population Sciences (IIPS), Ministry of Health and Family Welfare. National Family Health Survey 4 (2015-16): India Fact Sheet [Internet]. [cited 2020 Jun 5]. Available from: http://rchiips.org/NFHS/pdf/NFHS4/India.pdf

24. Indian Institute of Population Sciences (IIPS), Ministry of Health and Family Welfare. National Family Health Survey 4 (2015-16): India Report [Internet]. [cited 2020 Jun 5]. Available from: http://rchiips.org/NFHS/NFHS-4Reports/India.pdf

25. Thankappan KR, Shah B, Mathur P, Sarma PS, Srinivas G, Mini GK, et al. Risk factor profile for chronic non-communicable diseases: results of a community-based study in Kerala, India. Indian J Med Res. 2010 Jan;131:53–63.

26. Sugathan TN, Soman CR, Sankaranarayanan K. Behavioural risk factors for non communicable diseases among adults in Kerala, India. Indian J Med Res. 2008 Jun;127(6):555–63.

27. Thakur J, Jeet G, Nangia R, Singh D, Grover S, Lyngdoh T, et al. Non-communicable diseases risk factors and their determinants: A cross-sectional state-wide STEPS survey, Haryana, North India. PLoS ONE [Internet]. 2019 Nov 27 [cited 2020 May 9];14(11). Available from: https://www.ncbi.nlm.nih.gov/pmc/articles/PMC6881003/

28. Gupta Milan, Singh Narendra, Verma Subodh. South Asians and Cardiovascular Risk. Circulation. 2006 Jun 27;113(25):e924–9.

29. Newtonraj A, Murugan N, Singh Z, Chauhan RC, Velavan A, Mani M. Factors Associated with Physical Inactivity among Adult Urban Population of Puducherry, India: A Population Based Cross-sectional Study. J Clin Diagn Res JCDR. 2017 May;11(5):LC15–7.

30. United Nations, Department of Economic and Social Affairs. World Urbanization Prospects: The 2018 Revision (ST/ESA/SER.A/420) [Internet]. [cited 2020 Jun 5]. Report No.: ISBN: 978-92-1-148319-2. Available from: https://population.un.org/wup/Publications/Files/WUP2018-Report.pdf

31. Devarajan R, Prabhakaran D, Goenka S. Built environment for physical activity—An urban barometer, surveillance, and monitoring. Obes Rev. 2020 Jan 1;21(1):e12938.

32. Selvaraj K, Kar SS, Ramaswamy G, Premarajan KC, Saya GK, Kalidoss V. Clustering of cardiovascular disease risk factors – Syndemic approach: Is sit a time to shift toward integrated noncommunicable disease clinic? Indian J Public Health. 2019 Jul 1;63(3):186.

33. Luhar S, Timæus IM, Jones R, Cunningham S, Patel SA, Kinra S, et al. Forecasting the prevalence of overweight and obesity in India to 2040. PLOS ONE. 2020 Feb 24;15(2):e0229438.

34. Ministry of Commerce and Industry. Fresh Fruits and Vegetables Production in India [Internet]. [cited 2020 May 26]. Available from: http://apeda.gov.in/apedawebsite/six_head_product/FFV.htm

35. Indian Institute of Population Sciences (IIPS), Ministry of Health and Family Welfare. National Family Health Survey 3 (2005-06): India Report [Internet]. [cited 2020 Jun 5]. Available from: http://rchiips.org/NFHS/nfhs3_national_report.shtml

36. Anchala R, Kannuri NK, Pant H, Khan H, Franco OH, Di Angelantonio E, et al. Hypertension in India: a systematic review and meta-analysis of prevalence, awareness, and control of hypertension. J Hypertens. 2014 Jun;32(6):1170–7.

37. Mohan V, Deepa M, Farooq S, Datta M, Deepa R. Prevalence, awareness and control of hypertension in Chennai--The Chennai Urban Rural Epidemiology Study (CURES-52). J Assoc Physicians India. 2007 May;55:326–32.

38. Indian Council of Medical Research, Public Health Foundation of India, Institute for Health Metrics And Evaluation. India: Health of the Nation's States (The India State-Level Disease Burden Initiative) [Internet]. Indian Council of Medical Research, Public Health Foundation of India, and Institute for Health Metrics and Evaluation; 2017. Report No.: ISBN 978-0-9976462-1-4. Available from: http://www.healthdata.org/sites/default/files/files/2017_India_State-Level_Disease_Burden_Initiative_-_Full_Report%5B1%5D.pdf

39. International Diabetes Federation. International Diabetes Federation Diabetes Atlas 8th Edition 2017 [Internet]. [cited 2020 May 26]. Report No.: ISBN: 978-2-930229-87-4. Available from: https://www.idf.org/e-library/epidemiology-research/diabetes-atlas/134-idf-diabetes-atlas-8th-edition.html

40. Nanditha A, Snehalatha C, Satheesh K, Susairaj P, Simon M, Vijaya L, et al. Secular TRends in DiabEtes in India (STRiDE–I): Change in Prevalence in 10 Years Among Urban and Rural Populations in Tamil Nadu. Diabetes Care. 2019 Mar 1;42(3):476–85.

41. Joshi SR, Anjana RM, Deepa M, Pradeepa R, Bhansali A, Dhandania VK, et al. Prevalence of dyslipidemia in urban and rural India: the ICMR-INDIAB study. PloS One. 2014;9(5):e96808.

42. Oommen AM, Abraham VJ, George K, Jose VJ. Rising trend of cardiovascular risk factors between 1991–1994 and 2010–2012: A repeat cross sectional survey in urban and rural Vellore. Indian Heart J. 2016 May 1;68(3):263–9.

43. Dandona L, Dandona R, Kumar GA, Shukla DK, Paul VK, Balakrishnan K, et al. Nations within a nation: variations in epidemiological transition across the states of India, 1990–2016 in the Global Burden of Disease Study. The Lancet. 2017 Dec 2;390(10111):2437–60.

44. Khorrami ZM, Etemad K, Yarahmadi S, Khodakarim S, Kameli M, Hezaveh AM, et al. Urbanization and noncommunicable disease (NCD) risk factors: WHO STEPwise Iranian NCD risk factors surveillance in 2011. East Mediterr Health J Rev Sante Mediterr Orient Al-Majallah Al-Sihhiyah Li-Sharq Al-Mutawassit. 2017;

45. Ministry of Health and Family Welfare. National multisectoral action plan for prevention and control of common noncommunicable diseases (2017-2022) [Internet]. [cited 2020 Jun 5]. Available from: https://main.mohfw.gov.in/sites/default/files/National%20Multisectoral%20Action%20Plan%20%28NMAP%29%20for%20Prevention%20and%20Control%20of%20Common%20NCDs%20%282017-22%29_1.pdf

46. National Health Authority. Annual Report: Ayushman Bharat (Pradhan Mantri Jan Arogya Yojana) [Internet]. [cited 2020 Jun 5]. Available from: https://www.pmjay.gov.in/sites/default/files/2019-09/Annual%20Report%20-%20PMJAY%20small%20version_1.pdf

47. Yadav K, Krishnan A. Changing patterns of diet, physical activity and obesity among urban, rural and slum populations in north India. Obes Rev Off J Int Assoc Study Obes. 2008 Sep;9(5):400–8.

48. Armstrong T, Bull F. Development of the World Health Organization Global Physical Activity Questionnaire (GPAQ). J Public Health. 2006 Apr 1;14(2):66–70.
