# Supplementary material for: Profile of risk factors for Non-Communicable Diseases (NCDs) in a highly urbanized district of India: Findings from Puducherry district-wide STEPS Survey, 2019–20
Source: PLoS One. 2021 Jan 12;16(1):e0245254. doi: 10.1371/journal.pone.0245254 (PMC7802941; doi:10.1371/journal.pone.0245254)
Supplement: Attachment — Submitted filename: Response to reviewers.docx [file pone.0245254.s002.docx]

**Rebuttal letter**

Please find point wise response to the queries raised by the reviewers. We thank you for helping us to improve our manuscript.

**Reviewer 1**

**Comment: Line 81: what is 9.8 lakhs?**

**Author response:** The population of Puducherry district is 9.8 Lakhs. To suit the international readers, the population size has been converted into millions (0.98 million) in the manuscript. **(Line no: 80)**

**Comment: You should indicate the exclusion criteria**

**Author response:** Exclusion criteria have been included in the manuscript

**(Line number: 104-107)**

**Comment: Did the WHO STEPS team receive a copy of the STEP instrument after adaptation and before finalization as recommended?**

**Author response:** As this study was a district level survey (small scale survey), the WHO STEPS team was not contacted after adaptation and before finalization of the study tool. However, the final study tool has been reviewed for its scientific rigor and was approved by the ethics committee of the institute before beginning the study.

**Comment: Regarding the recruitment and training of staff: specify the number of field team members and briefly explain the training of data collectors**

**Author response:** Information about recruitment and training of staff has been included. **(Line number: 144-147)**

**Comment: Line 141: specify the room and location chosen for the collection of blood samples in the STEP 3 procedure**

**Author response:** The location chosen for collection of blood samples has been included.

**(Line no: 150-151)**

**Reviewer 2**

**Comment: Please specify the year of study in the title**

**Author response:** The year of study has been specified in the title of the study

**(Line number: 4)**

**Comment: Specify the name of the institute to which the ethics committee belongs**

**Author response:** The institute to which the ethics committee belongs has been included in the manuscript **(Line numbers: 122-123)**

**Comment: Unless I'm mistaken, I don't see the survey rate**

**Author response:** The survey response rate is already present in the results section.

**(Line numbers: 198-199)**

**Comment: Refer in the methodology to a study or studies that describe the STEPS protocol in detail**

**Author response:** References to studies that describe STEPS protocol in detail have been included in the methodology section. **(Line number: 91)**

**Comment: Bibliographic references are too old (in yellow in the attachment)**

**Author response:** Highlighted bibliographic references that were old (before the year 2010) have been removed. But, references of cardinal studies such as BMI classification for Asian population, development of WHO GPAQ scale, Kish method and other key research papers were retained in the bibliography.

**Comment:** **Your discussion appears too vague. the discussion is not synthetic enough. Couldn't you synthesize the different step studies from the different regions of India in a table or a figure?**

**Author response:** Key results of various STEPS studies from different regions of India have been synthesized and presented as a table in the manuscript **(Line number: 402)**

**Comment: Items can be deleted (in blue in the attachment). It may be necessary to broaden to a public health dimension i.e. recommendations or possible actions in reference to WHO diabetes policies, India, etc...**

**Author response:** Items highlighted in blue have been deleted. The discussion section has been broadened by incorporating various national and international programs and policies for prevention and control of NCDs. **(Line numbers: 356-391)**

**Comment: Briefly broaden the discussion to include other STEP studies published internationally.**

**Author response:** Discussion section has been broadened to include various STEPS studies published internationally. **(Line numbers: 247-253, 265-267, 282-284, 288-293, 307-309, 316-318, 328-330)**

**In addition to changes suggested by the reviewers, minor modifications have also been suggested by the corresponding and other authors of the manuscript while they reviewed the manuscript. Such changes have also been highlighted in manuscript for kind perusal of the reviewers.**
